# Participant and facilitator perspectives on a psychoeducational intervention for individuals at risk of bipolar disorder

**DOI:** 10.1192/bjo.2025.10856

**Published:** 2025-10-07

**Authors:** Heeva Chavoshi Nejad, Emma Morton, Clara Westwell-Roper, John-Jose Nunez, Alexander Levit, Ashley Forbes, Tera Armel, Erin E. Michalak, Eduard Vieta, Lakshmi N. Yatham, Kamyar Keramatian

**Affiliations:** Faculty of Law, McGill University, Montreal, Quebec, Canada; School of Psychological Sciences, Monash University, Clayton, Australia; Department of Psychiatry, University of British Columbia, Vancouver, British Columbia, Canada; Vancouver Coastal Health Authority, Vancouver, British Columbia, Canada; Faculty of Health Sciences, Queen’s University, Kingston, Ontario, Canada; Department of Psychiatry and Psychology, Hospital Clinic, Institute of Neuroscience, University of Barcelona, IDIBAPS, CIBERSAM, Barcelona, Catalonia, Spain

**Keywords:** Bipolar disorder, psychoeducation, high risk, qualitative study, programme evaluation

## Abstract

**Background:**

Bipolar disorder often goes unrecognised for several years, leading to delayed treatment and negative outcomes. To help address this, we have developed a novel telehealth-based group psychoeducational and resilience enhancement programme for individuals at high risk for bipolar disorder (PREP-BD), aimed at improving help-seeking among adolescents and young adults at risk of developing bipolar disorder.

**Aims:**

The purpose of the current study was to explore the perspectives of at-risk youth, their families and group facilitators who participated in the feasibility trial of PREP-BD.

**Method:**

Group and individual semi-structured feedback sessions were conducted with the participants (*n* = 21) of the programme, their family members and the facilitators of PREP-BD. The questions covered their experiences, opinions on the programme’s structure and content and suggestions for improvement. Feedback sessions were transcribed and analysed qualitatively using inductive content analysis.

**Results:**

Overall feedback was positive, with participants and facilitators appreciating the informative and engaging nature of the sessions. Some participants desired more actionable resources and complex content. Family members sought greater involvement and information about the programme. The online format was valued for convenience, but was also viewed as a barrier by some to fostering deeper connections.

**Conclusions:**

PREP-BD shows promise as a psychoeducational intervention for individuals at high risk for bipolar disorder. To enhance the programme’s effectiveness, future iterations should incorporate more nuanced content, provide additional practical guidance and address the limitations of the virtual setting. Continued evaluation and optimisation are crucial for ensuring the programme’s effectiveness as a tool for early intervention in bipolar disorder.

Bipolar disorder often remains undiagnosed for a multitude of years.^1–3^ There is an urgent need to facilitate help-seeking for those at high risk of developing bipolar disorder to reduce this diagnostic delay and its associated serious consequences, including higher rates of suicide than the general population,^4^ severe difficulties with interpersonal relationships^2^ and increased hospitalisations for those untreated over 8 years.^5^ The delay in diagnosing bipolar disorder is a complex phenomenon influenced by various factors related to the illness (e.g. early depressive episodes often occur years before the first [hypo]manic episode, delaying recognition of bipolar disorder), the patient (e.g. underreporting of manic symptoms leading to misdiagnosis) and healthcare systems (e.g. limited specialist access and brief primary care visits).^6^ Among these factors are also patients’ lack of awareness of the early signs of bipolar disorder, as well as self-stigmatisation of bipolar disorder, which can result in delays in help-seeking.^1,6^

Psychoeducation is an intervention with the potential to address these delays by supporting individuals with bipolar disorder to learn about their condition, its treatment and self-management strategies to detect and prevent relapses.^7^ Such skills and knowledge are also relevant to the at-risk group. However, despite their strong evidence base,^8,9^ existing psychoeducation programmes are not specifically tailored to meet the unique needs of those at high risk for bipolar disorder. For instance, targeted information on modifiable risk factors (e.g. substance use, sleep hygiene) may mitigate the risk of illness onset for the at-risk population. Better awareness of, and less stigma about, depression and mania may also encourage earlier help-seeking and more informed and open discussions with clinicians before a diagnosis is made. Such potential benefits require investigation. As such, we have developed a novel telehealth-based group psychoeducational and resilience enhancement programme (PREP-BD) for individuals at high risk for bipolar disorder, aimed at reducing stigma and improving help-seeking among adolescents and young adults at risk of developing bipolar disorder. To identify individuals at high risk of developing bipolar disorder, this study utilised the bipolar at-risk (BAR) criteria. Studies using BAR criteria show that 10–23% of at-risk individuals progress to bipolar disorder within 1–2 years,^10^ rising to 29% over 11 years,^11^ highlighting the need for early intervention.

Findings from our feasibility trial demonstrated strong feasibility, with a 100% sign-up rate of individuals deemed eligible to participate and a 76.19% completion rate (attendance at ≥75% of sessions).^12^ The study indicated improvements in help-seeking intentions, particularly in response to a hypomanic scenario, as well as significant improvements in quality of life. While resilience and self-stigma showed non-significant trends toward improvement, these findings support the potential of psychoeducation in fostering early intervention efforts. Future randomised controlled trials are needed to further assess PREP-BD’s effectiveness.^12^ The current study aimed to qualitatively explore the perspectives of at-risk youth, their families and group facilitators who participated in the feasibility trial of PREP-BD, to refine the programme’s content and delivery methods.

## Method

### Design

The intervention in the PREP-BD feasibility study comprised 8 weekly, 60-min group psychoeducation sessions conducted via Zoom. Family members were invited to join for the screening session and final psychoeducation session. Participants were assigned to one of four cohorts. The topics covered, including depression, substance use, treatment of bipolar disorder,coping strategies and hypomania/mania, were adapted from the *Psychoeducation Manual for Bipolar Disorder* by Colom et al.^13^ While the original manual was designed for individuals with an established diagnosis of bipolar disorder, it was modified for a high-risk population by K.K. (assistant professor in psychiatry who is specialised in early intervention in bipolar disorder) and edited by E.M. (senior lecturer and psychologist specialised in psychosocial interventions in bipolar disorder). The main modifications involved shifting from a 21-session format focused on treatment adherence and pharmacotherapy for diagnosed individuals to a shorter, online programme centred on raising awareness of the early signs of bipolar disorder and reducing stigma in those not yet diagnosed. E.V., who co-authored the original psychoeducational manual, approved these changes. An overview of the content can be found in Table 1.

**Table 1:** Psychoeducational and resilience enhancement programme for individuals at high risk for bipolar disorder, session content

Session no.	Focus	Tasks
1	Group formation	Developing rapport; discussing the purpose of the intervention; establishing the rules intended to create a safe environment for participation; helping participants identify their individual goals
2	What is bipolar disorder?	Introducing the biopsychosocial model of bipolar disorder and dispelling the numerous myths about the illness; discussing the genetic and environmental risk factors; differentiating between normal mood variations and episodic mood changes associated with bipolar disorder; developing a daily mood and sleep diary
3	Mania and hypomania	Reviewing signs and symptoms of (hypo)mania; discussing potential triggers and early signs of relapse; helping participants develop an individualised intervention plan
4	Depression and suicide	Reviewing signs and symptoms of depression; discussing the risk of suicide; helping participants develop an individualised safety plan
5	Resilience building and stress management	Discussing resilience building and stress management strategies, such as creating a balanced schedule, regular exercise routine, social engagement, problem solving, proper sleep hygiene and breathing exercises
6	Treatment of bipolar disorder	Providing an overview of different classes of psychotropic medications; discussing the risk of ‘manic switch’ with antidepressants
7	Substance use	Reviewing the complex relationship between substance use and bipolar disorder; encouraging open and non-judgemental discussion about recreational substances
8	Closure (family members are welcome)	Encouraging participants to ask questions about the materials presented in the previous sessions; discussing community resources for patients and families

The feasibility evaluation of PREP-BD took place between September 2023 and April 2024. Following completion of the feasibility trial, feedback sessions were conducted with participants, family members and facilitators to gather their insights and suggestions for improvement on PREP-BD. Feedback sessions took place between November 2023 and May 2024.

The authors assert that all procedures contributing to this work comply with the ethical standards of the relevant national and institutional committees on human experimentation, and with the Helsinki Declaration of 1975 as revised in 2013. All procedures involving human subjects/patients were approved by the UBC Clinical Research Board Ethics on 12 December 2022 (ID: H22-01060).

### Participants and family members

Those eligible to participate in the PREP-BD feasibility study were individuals aged between 15 and 24 years and who fulfilled at least one of the BAR criteria^10^ within the previous 12 months: (a) a history of subthreshold mania; (b) a history of depression as well as cyclothymic features; and (c) a history of depression as well as a first-degree relative with bipolar disorder. Research suggests that predictive accuracy is highest when genetic and clinical risk factors are considered together.^14^ The BAR criteria reflect this approach and are widely used to identify individuals at high risk for bipolar disorder.^11^ Participants were recruited through advertisements placed in local youth clinics, as well as online platforms including Reddit, UBC’s Paid Participants Studies List and Reach BC. Recruitment took place from June 2023 to February 2024. Documentation of informed consent was obtained virtually via UBC REDCap following completion of the phone screening and prior to the clinical screening session, where K.K. confirmed their full eligibility using a combination of a structured interview and unstructured clinical interview incorporating the operationalised BAR criteria. For participants under the age of majority who were deemed incapable by K.K. of providing informed consent, parental or guardian consent was obtained and assent was secured from the participant following a review of the study’s purpose, procedures, risks and assent form. Twenty-one participants (15 female, 4 male and 2 non-binary, mean age 19.95 years, s.d. 2.04) took part in the PREP-BD feasibility intervention, 6 of them having invited their family members (3 were mothers, 1 was a father and 2 were siblings) to participate in the final intervention session and a family feedback session. Demographic information is provided in Table 2. Sixteen participants completed the intervention, defined as attending six out of eight sessions. All participants, including non-completers, took part in this qualitative study. Four of the six family members took part in individual feedback sessions.

**Table 2:** Participant and family member demographics and cohort

Participant number	Gender	Age	Ethnicity	Cohort number	Sessions attended	Status
1	F	19	White	1	8/8	Completer
2	M	22	Asian	1	6/8	Completer
3	M	20	Asian	1	8/8	Completer
4	F	22	Asian	1	8/8	Completer
5	F	19	White	2	7/8	Completer
6	F	23	Asian	2	6/8	Completer
7	F	18	Mixed	2	7/8	Completer
8	F	23	White	2	8/8	Completer
9	NB	18	White	2	8/8	Completer
10	F	18	Asian	3	8/8	Completer
11	F	19	Asian	3	7/8	Completer
12	M	18	Asian	3	8/8	Completer
13	F	18	White	3	7/8	Completer
14	F	17	White	3	7/8	Completer
15	M	17	Mixed	3	2/8	Non-completer
16	F	23	Latinx	4	8/8	Completer
17	F	21	White	4	7/8	Completer
18	F	22	White	4	5/8	Non-completer
19	NB	21	White	4	4/8	Non-completer
20	F	21	Asian	4	3/8	Non-completer
21	F	20	White	4	5/8	Non-completer
22	F	52	Asian	1	N/A	Family member
23	F	Not reported	White	2	N/A	Family member
24	M	44	White	3	N/A	Family member
25	F	60	White	4	N/A	Family member

M, male; F, female; NB, non-binary; N/A, not available.

### Facilitators

The facilitation team included four mental healthcare professionals: A.F., a registered social worker; C.W.-R., a child and adolescent psychiatry fellow; A.L., a senior psychiatry resident; and J.-J.N., a psychiatrist specialising in mood disorders. Each facilitator led one cohort of participants through the intervention.

### Qualitative feedback sessions

Within the month following each cohort’s final PREP-BD session, a semi-structured feedback session was held with participants to gauge their opinions of the programme, with the goal of optimising future iterations of the intervention. The feedback sessions were conducted via video-conferencing and were audio-taped for transcription. A research team member (H.C.N.) moderated the sessions using a set of pre-written questions that focused on participants’ opinions and experiences with PREP-BD (Table 3). Although our intent was to have all participants of each cohort attend a single group feedback session, this was sometimes not possible due to scheduling conflicts; in these cases, feedback was sought in one-to-one sessions (Table 4). A separate feedback session was scheduled for the family members participating in each cohort. The feedback session for the facilitators was then led by a research team member (K.K.) to gather their perspectives on the intervention, the challenges they faced and their observations on the participants’ engagement and outcomes.

**Table 3:** Semi-structured feedback session questions

Participant feedback session questions
What was your overall experience of the programme? What was the most helpful part of participating in the programme? Was there anything about participating in the programme that you found unhelpful? How did you find the video-conferencing format of the groups? What do you think about the length of the sessions? How about the number of sessions? Was there any session or topic that you did not find helpful or necessary? Was there any topic that you had expected or hoped to see covered in the programme but wasn’t? How did you find the handouts given at the end of each session? Was there anything that the facilitator did that you found particularly helpful? Is there anything they could improve on? Is there anything else we should have talked about today but didn’t?
Family feedback session questions
What was your general impression of the programme? What was the impact of the programme on your child/partner? What do you see as the most helpful part of your child/partner participating in the programme? What do you see as the most unhelpful part of your child/partner participating in the programme? Were you satisfied with the level of your involvement in the programme? Was there anything else you would like to see done to support carers as a part of this programme? Is there anything else we should have talked about today but didn’t?
Facilitator feedback session questions
What was your overall experience of facilitating the sessions? What do you see as the most helpful part for participants in the programme? What do you see as the most unhelpful part for participants in the programme? How did you find the video-conferencing format of the groups? What was your experience with participants having their cameras on or off? Should we make having them on mandatory? What do you think about the length of the sessions? How about the number of sessions? Did you find the facilitator manual easy or difficult to adhere to? What are your thoughts on the interactive portions of the sessions? Is there anything else we should have talked about today but didn’t?

**Table 4:** Attendees of each cohort’s feedback session^a^

Interviewee	Number of attendees	Length of feedback session (min:s)
Cohort 1 participants	4	12:11
Cohort 2 participants; feedback session 1	3	25:13
Cohort 2 participants; feedback session 2	1	5:04
Cohort 2 participants; feedback session 3	1	4:26
Cohort 3 participants; feedback session 1	5	30:21
Cohort 3 participants; feedback session 2	1	10:18
Cohort 4 participants; feedback session 1	2	19:21
Cohort 4 participants; feedback session 2	2	13:20
Cohort 4 participants; feedback session 3	1	8:45
Cohort 4 participants; feedback session 4	1	7:35
Cohort 1 family members	1	27:55
Cohort 2 family members	1	6:13
Cohort 3 family members	1	7:09
Cohort 4 family members	1	8:18
Facilitators	4	61:52

a. Though our intent was to have all participants of each cohort attend a single group feedback session, this was sometimes not possible due to scheduling conflicts. In these cases, feedback was sought in one-to-one sessions.

### Data analysis

The collected data were transcribed verbatim, but are presented below as a naturalised transcription where non-essential filler words and hesitations have been removed to enhance clarity.^15,16^ Using NVivo Pro version 14.0 for macOS (Lumivero, Denver, Colorado, USA; https://download.qsrinternational.com/Software/NVivo14forMac/NVivo.dmg), H.C.N. analysed the transcripts using inductive content analysis; these were then reviewed and coded independently by H.C.N. Inductive content analysis followed a systematic approach, including several key steps: familiarisation with the data by reading and re-reading transcripts; generation of initial high-level codes on major topics of interest (known as ‘chunking’ or big picture coding); development of granularity in coding by searching for subcategories and fine-grained codes; refinement and review of codes and categories; and interpretation of the data collected through a narrative synthesis of findings.^17^

To enhance the rigour of the analysis, the categories were verified through member checking with a research team member who has lived experience with bipolar disorder.^18^ Additionally, regular debriefing sessions were held with co-authors E.M. and K.K. throughout the analytic process, where the codes were reviewed and discussed to ensure that results accurately reflected participants’ experiences.

## Results

Feedback sessions with participants lasted an average of 13.7 min (range 4.4–30.5 min); family feedback sessions lasted an average of 12.4 min (range 6.2–27.9 min), and the average facilitator feedback session was 61.9 min. For detailed participant demographic information and the composition of each group, refer to Table 2.

### Overview of key findings

General positive feedback on the experience and impacts of participating in PREP-BD is presented below. Specific aspects of the programme that received feedback, whether positive or negative, are addressed in the relevant sections below. Overall feedback about the programme was largely positive, with participants expressing satisfaction with PREP-BD. Many reported enjoying being a participant, with participant no. 15 describing it as ‘a nourishing experience’. Facilitators also provided positive feedback, highlighting that their overall impression of facilitating the programme was a ‘really positive experience’ (A.F.). Family members highlighted the programme’s beneficial impact on their loved ones. They appreciated the participants’ motivation and engagement, with one mother noting that it was ‘good that [her son] showed interest and participated’ (family member no. 22). The programme was viewed as valuable in helping participants gain a deeper understanding of themselves. One family member noted this about her daughter: ’She’s learning a lot about herself and her disorder’ (family member no. 23). To clarify, the daughter was high risk for bipolar disorder but undiagnosed, which had been explained to the family member when discussing the study’s objectives via email and the clinical screening session.

Specific feedback about aspects of the programme related to the research aim was grouped into three categories: ‘group dynamics’, ‘programme content’ and ‘programme structure’, with additional subcategories highlighting important trends in feedback on these topics (Table 5). Category summaries are presented below, with quotes that reflect each category.

**Table 5:** Categories and subcategories identified through analysis of the qualitative feedback sessions

Category	Group dynamics	Programme content	Programme structure
**Subcategory**	Inter-participant relationships	Familiarity with information	Family’s desire for more involvement
	Relationship with facilitator	Presence of actionable resources	Benefits of online format
		Desire for more nuanced information	Challenges of online format
		Relevance of pharmacology content	

### Group dynamics

The category of group dynamics captured feedback related to three subcategories: inter-participant relationships, relationship with facilitator and programme content.

#### Inter-participant relationships

This subcategory encompasses how interactions and relationships among participants affected their overall programme experience. Participants expressed appreciation for PREP-BD’s facilitation of peer bonding through group discussions. One participant noted: ‘The most helpful would probably be getting to know everyone and feeling encouraged to actively be part of the conversation’ (participant no. 16). Many participants valued the opportunities for discussion because these allowed the fostering of solidarity and mutual support among group members. Some participants desired more group discussions to strengthen cohort bonds: ‘I wish we had more time to discuss with each other and be more connected as a group’ (participant no. 13). Both participants and family members highlighted the benefit of connecting with others through the programme who could relate to their experiences. For instance, one participant noted: ‘Just having a chance to hear other people’s experiences makes you feel more validated in your own experiences’ (participant no. 20). However, this same participant suggested that increased group cohesion could improve comfort in sharing: ‘If there is more group bonding, it would have just made us more comfortable to share more’. Specific aspects of the programme that facilitated interaction between cohort members were appreciated, including the Jamboards (a digital whiteboard used for real-time collaboration), icebreakers and group discussions.

#### Relationship with facilitator

This subcategory focuses on participants’ interactions with the facilitators, and how these interactions impacted their engagement and satisfaction with the programme. Participants valued the facilitators’ ability and willingness to answer their questions. Participant no. 18 remarked: ‘[The facilitator] was really open to answering questions, and I found that was really helpful for understanding the material and how it would apply to me’. Having knowledgeable facilitators was also emphasised as being valuable: ‘Knowing that the information we see in the sessions are from a professional – I think it feels a lot more valid to me, and I can trust that information more’ (participant no. 10). Participant no. 16, part of a cohort led by a non-physician facilitator, voiced a desire for a physician to join a session, noting that they had ‘lots of questions that we had that weren’t fully answered’. Additionally, participants valued the facilitators’ efforts to create a safe and comfortable environment, which encouraged them to share their experiences. Participant no. 15 commented that their cohort’s facilitator ‘encouraged [the participants] to share anything, no right or wrong answers, and I felt like that was very encouraging for me and many others’.

### Programme content

The category of programme content captured feedback related to four subcategories: familiarity with information, presence of actionable resources, desire for more nuanced information and relevance of pharmacology content.

#### Familiarity with information

Several participants noted that a portion of the programme’s content was not new to them. As participant no. 7 articulated: ‘Sometimes the information felt a bit redundant, and sometimes it felt a bit shallow because it was very surface-level understanding’. Other participants concurred, describing the programme content regarding stress management as ‘obvious’ (participant no. 17) and ‘basic’ (participant no. 20). Certain family members and facilitators shared similar sentiments. Multiple participants noted that content relating to self-help, specifically, came across as didactic. Participant no. 19 explained that discussions on this topic were ‘a little bit patronizing … it was kind of like exercise, eat, good sleep. The things that kind of like everyone already knows to do, but that get really hard to do when you’re trying to manage mental illness’.

#### Desire for more nuanced information

Participants described wanting more complex content within the programme. Multiple participants also conveyed a desire to learn about how the information presented related to their experience with mental health specifically. Participant no. 20 commented: ‘I felt [the program’s content] got generalized on a lot of occasions’. Facilitators observed that the programme’s discussions on substance use were overly focused on abstinence rather than harm reduction, with C.W.-R. noting ‘a real need to … make a safe space for discussion to talk about use … with a harm reduction approach’.

#### Relevance of pharmacology content

Participant and facilitator feedback on the pharmacology content of the programme diverged. Many participants found the pharmacology session less relevant to their needs. For example, participant no. 11 asserted: ‘… the pharmacology session was a bit too overwhelming … there were a bit too many terms that we would not remember’. Others wished that this content had been expanded upon, with participant no. 16 specifying a desire to learn about practical information on side-effects and managing medication rather than medication types. Some facilitators believed that pharmacology content could validate mental illness as a biological construct, and suggested adding more data on medication efficacy and side-effects. C.W.-R. stated: ‘I think [participants] might benefit from … some data on [whether] people get better when they take medications versus not’ in order to ‘legitimize the idea of pharmacotherapy’. Other facilitator-suggested that additions were strategies for self-advocacy regarding finding the right medication.

#### Presence of actionable resources

Participants noted the value of the tangible and practical tools provided in the programme, such as the mood chart and self-rated mania scale. Participant no. 18 remarked: ‘It was good to have some appliable skills. Like the mood chart, especially, I thought was really great’. One family member also noted these actionable resources to be the most helpful part of the programme for participants in their opinion, further expressing that their loved one ‘learned quite a bit, and she was actually surprised with how much she was able to take home’ from the sessions (participant no. 24). However, there was also a desire for additional resources and detailed guidance on seeking professional help: ’Something that I was surprised wasn’t included was more concrete steps or talking about the logistics of seeking help’ (participant no. 4).

### Programme structure

The category of programme structure captured feedback related to three subcategories: the family’s desire for more involvement, benefits of an online format and challenges of an online format.

#### Family’s desire for more involvement

This subcategory highlights family members’ requests for greater engagement with, and more information about, the programme. Multiple interviewees expressed this wish, with family member no. 24 noting: ‘I think I was hoping that I could have a bit more involvement’. Several sought more detailed information about the programme’s goals and content, with comments such as: ‘I would love to have more information from the beginning’ (family member no. 23). There were also suggestions for additional parent-focused components, including separate sessions and recorded materials for those unable to attend.

#### Benefits of online format

This subcategory highlights the advantages of conducting the programme via video-conferencing. The convenience of Zoom sessions was highly valued by participants, because it allowed them to attend from a location of their choosing. Participant no. 5 appreciated the flexibility, noting: ‘I liked the Zoom format … being in-person would have been a hindrance as oftentimes I don’t have the energy to leave my home’. Additionally, the online format provided comfort, as participants felt more at ease joining the sessions online. As participant no. 15 remarked: ‘It just felt like a close, private format which I did like’, emphasising how this ‘encouraged [him] to speak or … feel comfortable’. Participant no. 10 appreciated having the choice ‘to share and … to turn [her] video on or not’ because of the online format. Similarly, participant no. 20 voiced feeling ‘very comfortable having [her] camera off and not talking’.

#### Challenges of online format

In this subcategory, the difficulties associated with the online format concerning both facilitators’ and participants’ experiences are addressed. Facilitators who led groups where cameras were generally turned off noted that this impacted their overall enjoyment of the experience. A.L. mentioned that it is ‘a drag staring at a blank screen’, and C.W.-R. concurred, stating that it is ‘less enjoyable as a facilitator when people choose not to have cameras on’. The anonymity provided through having cameras off also raised concerns from C.W.-R. about ensuring confidentiality, because facilitators were unable to visually confirm who was present or whether participants were alone. Some participants reported that reluctance to turn on cameras in the virtual setting made it harder to build group connection, an aspect of the programme emphasised in the inter-participant relationships theme. For instance, participant no. 11 shared that: ‘it’s hard to engage because … nobody else has their cameras on, so I feel like I can’t really connect with people’. In a similar vein, noting that the lack of physical presence made it harder to build deeper relationships and engage in meaningful discussions, participant no. 12 voiced: ‘I feel like the program would be run better as an in-person thing because it allows for more discussion and more connectedness’. Participant no. 12 also noted that factors such as session timing impacted camera use, explaining that he would ‘love to come and turn on [his] camera, but … at 8:00 pm … [he does] not have the energy to be on camera’. Participant no. 14 agreed, stating: ‘we all shared pretty similar sentiments of, you know, it’s late … don’t really want to show up fully [inaudible] and turn my camera on’.

## Discussion

This qualitative study aimed to explore the experiences and perspectives of participants, their families and group facilitators involved in the feasibility trial of using PREP-BD, a novel psychoeducational programme, in adolescents and young adults who are at risk of developing bipolar disorder. The positive feedback regarding the programme’s content, structure and delivery indicates that the PREP-BD psychoeducational intervention is often well received by participants, family members and facilitators. The psychoeducational content was found to be informative, straightforward and engaging, fulfilling one of the programme’s primary objectives: to enhance participants’ understanding of bipolar disorder. Participants and facilitators also highlighted several challenges and suggestions for improvement, which will be valuable in refining future versions of the programme.

The programme’s group format emerged as a significant strength, fostering a sense of community and mutual support among participants. This finding aligns with other research that identified the sharing of lived experiences as a crucial component of successful group psychoeducation for individuals with an established diagnosis of bipolar disorder.^18,19^ Conversely, participants of an individualised psychoeducation programme for high-risk psychosis critiqued the lack of a group format, expressing a desire to share experiences with others.^20^ Evidence from a quantitative meta-analysis supports the view that psychoeducation delivered in a group or family setting is more beneficial than individual formats, particularly in reducing the recurrence of mood episodes.^9^ Our results suggest that the benefits of peer support in group settings may also extend to those at high risk for bipolar disorder. The opportunity to connect with others facing similar challenges probably provided participants with a sense of validation and reduced feelings of isolation – key factors identified by both Weiner et al^18^ and O’Connor et al^21^ as contributing to patient satisfaction in group psychoeducation for bipolar disorder. Future iterations of PREP-BD will capitalise on this strength, integrating additional opportunities for participant interaction. Furthermore, although the qualitative analysis did not reveal clear cohort effects, facilitators, upon sharing their experiences during the feedback session, recognised that some cohorts appeared more engaged and willing to participate than others. While these observations were anecdotal, future studies could systematically assess cohort differences by statistically comparing group cohesion scores or exploring the influence of facilitator characteristics in a larger, adequately powered sample.

The favourable feedback on facilitator performance underscores the importance of skilled facilitation in creating a supportive and engaging learning environment. Facilitators were praised for their ability to foster open communication, answer questions effectively and create a safe space for participants to share their experiences. Participants in Poole et al’s^19^ study regarding group psychoeducation for bipolar disorder also noted an appreciation for facilitators who avoided an authoritative tone or lecture-like approach to facilitating, which helped foster an environment where participants felt valued and important. This consistent finding underscores the importance of facilitators’ interpersonal skills in psychoeducation programmes for bipolar disorder. Participants also emphasised the importance of knowledgeable facilitators, whose expertise fostered trust and confidence in the information provided. Trusted facilitators in psychoeducational programmes can address barriers to help-seeking by offering credible guidance,^19,21–23^ a critical need because youth with bipolar disorder may struggle to find reliable information online.^24^

Feedback revealed several areas where PREP-BD could be enhanced to better meet the needs of participants and their families. A key subcategory was the desire for more nuanced and complex content, with sessions on self-help strategies and substance use described as overly simple or patronising. This suggests that, while the programme may have successfully addressed the needs of those with less prior knowledge of bipolar disorder, it may not have fully engaged participants with more advanced understanding or specific informational needs. Similar to the present study, participants in that of Poole et al^19^ noted a specific appreciation of the content related to medication, but criticised the programme as a whole for being too medically orientated. However, in our study there were also participants and facilitators who voiced a desire for more medical-related content. These differing viewpoints suggest that future iterations of the programme may benefit from a more flexible approach that accommodates varying informational needs and levels of prior knowledge. Furthermore, adopting a less medicalised approach that combines essential information with participant-centred language could alleviate concerns about overwhelming content while still emphasising the role of medication in treatment. Other suggestions on how content could be strengthened included additional actionable resources and practical guidance, particularly in relation to seeking professional help and coping strategies for managing bipolar disorder. This feedback aligns with broader recommendations to incorporate skills training in psychoeducation, because these strategies enhance coping skills and help prevent recurrences more effectively than didactic lecturing.^9^ Additionally, it is vital that future adaptations of this psychoeducational intervention are updated to accurately reflect the resources and actionable guidance available to future participants in their locale.

Feedback regarding PREP-BD’s telehealth format was divided. On the one hand, it was appreciated by most participants for its convenience and anonymity, aligning with research on computerised cognitive behavioural therapy for depression, which showed that the anonymity of online self-help tools is a motivating factor encouraging users to consistently engage with the programme.^25^ Additionally, Poole et al^19^ found that bipolar disorder-related symptoms and feasibility issues were major barriers to attending in-person sessions, which participants in this study noted were easier to circumvent because of the online format’s flexibility. Nevertheless, online delivery presented some challenges; a few participants mentioned that the virtual format limited group cohesion. One participant linked this directly to other participants having their cameras off during the sessions. This aligns with Poole et al,^19^ where participants in in-person psychoeducation expressed the view that seeing each other in sessions was a key factor in building connections. In the current study, the option to keep cameras off may have disrupted this dynamic, reducing the sense of group engagement for these participants. However, several other participants valued the comfort and reduced pressure of being able to keep their cameras off. Facilitators, in particular, reported feeling disconnected from their cohort when cameras were turned off, making it harder for them to gauge participant engagement and affecting their experience in the programme. Even with video input, Thomas et al^26^ reported some studies in which therapists struggle more with reading non-verbal cues in virtual settings. These findings suggest that, while telehealth offers significant advantages, it also requires careful consideration of strategies to foster connection and engagement in a virtual environment for participants and facilitators alike. Future iterations of the PREP-BD programme will leverage recommendations from the online learning space to promote camera use, such as discussing the importance of non-verbal cues, impacts on group cohesion and the likelihood of improved instructor effectiveness.^27^

### Limitations

The findings of this study should be considered within the context of certain limitations. Despite H.C.N.’s probing, there was difficulty in getting participants to elaborate on their experiences, especially when asked yes/no questions. This limited our understanding of their feelings towards the programme and sometimes resulted in particularly short feedback sessions. Future feedback sessions may benefit from incorporating specific probes to investigate key aspects of participants’ experiences, and adopting exclusively open-ended questions may also elicit richer responses. Furthermore, given that group interviews with adolescents and young adults can be particularly challenging due to a reduced willingness to share openly in peer settings,^28^ incorporating introductory icebreakers or allowing time for open-ended, participant-led discussion at the start of the session may enhance participant engagement. Another limitation is that certain aspects of the programme, such as the individualised intervention plan, were neither discussed nor mentioned in the feedback sessions, leaving participant perspectives unknown.

The small sample size^29^ and geographic concentration of participants in Metro Vancouver may have limited the generalisability of the results to other populations or settings. Furthermore, with most of our participants being female, the prevalence of quotes from female participants may have introduced gender bias.^30^ While this trend of predominantly female participants is in line with the samples of related studies,^31–34^ it is worth noting that stigma is a powerful barrier to treatment engagement for young males.^35^ As a result, findings may primarily reflect the experiences of a highly engaged treatment-seeking group. Moreover, despite some advantages described above, the online format might not be suitable for all candidates for the intervention. Future research with a more diverse and representative sample could provide a more comprehensive understanding from the perspective of male and gender-diverse participants.

These limitations notwithstanding, PREP-BD shows significant promise as a psychoeducational intervention for individuals at high risk for bipolar disorder. Its ability to create a safe environment for those individuals, not only to learn more about the disorder but also to allow them to connect through lived experience, makes it auspicious as a valuable tool in mental health care. By addressing the areas identified for improvement – such as enhancing the depth of content and including more actionable resources – the programme can be further refined to better meet the needs of its participants. The benefits and drawbacks of conducting the study online, such as increased comfort versus lack of connection, must also be considered to ensure the programme’s utmost efficacy. Continued evaluation and optimisation of PREP-BD will be crucial for ensuring its effectiveness and sustainability as a tool for early intervention in bipolar disorder.

## Data Availability

The data that support the findings of this study are available from the corresponding author upon reasonable request.
